# The Inclusion of Black Cumin Meal Improves Growth Performance of Growing Awassi Lambs

**DOI:** 10.3390/vetsci7020040

**Published:** 2020-04-09

**Authors:** Belal S. Obeidat

**Affiliations:** Department of Animal Production, Faculty of Agriculture, Jordan University of Science and Technology, Irbid 22110, Jordan; bobeidat@just.edu.jo; Tel.: +962-2-7201000 (ext. 22214); Fax: +962-2-7201078

**Keywords:** Awassi lambs, black cumin meal, growth performance, nutrient digestibility

## Abstract

Twenty-four Awassi lambs were randomly divided into two dietary treatments to assess the influence of black cumin meal (BCM; *Nigella sativa* L.) feeding on growth performance. Diets were no BCM (CON) or 150 g BCM/kg DM (BCM150)). Lambs were fed the experimental diets for 80 days. Lambs were housed randomly in individual pens that were fitted with water and feed containers. During the study, nutrient intake was measured daily. Body weight (BW) and average daily gain (ADG) were evaluated biweekly. Nutrient digestibility and nitrogen (N) balance were evaluated on days 49–59. Intakes of dry matter, crude protein (CP), neutral detergent fiber (NDF), acid detergent fiber (ADF), ether extract (EE) and metabolizable energy were greater (*p* ≤ 0.05) in the BCM150 diet than in the CON diet. The digestibility of DM, CP and EE (*p* ≤ 0.04) was improved in the BCM150 diet compared to the CON diet. However, NDF and ADF digestibility did not differ between the two diets. Nitrogen retained (g/d; *p* = 0.01) and N retention (%; *p* = 0.05) was greater in the BCM150 compared with CON diet. Final BW, ADG, and feed efficiency (DM intake: kg of gain) was greater (*p* ≤ 0.002) in BCM150 lambs than the CON lambs. However, cost/kg of BW gain was lower in the BCM150 diet than in the CON diet. In conclusion, the inclusion of black cumin meal improved the growth performance and profits in diets of growing lambs. Therefore, it could be used as an alternative to soybean meal and barley as a protein and energy supplement, respectively.

## 1. Introduction

In Jordan, the sheep population is about 3.1 million [1], from which the Awassi is the predominate breed. Sheep are raised mainly for meat production, contributing to about 40% of the red meat consumption in Jordan [1]. Harsh environments and lack of good quality forages are the two major problems facing sheep producers in the Middle East, including Jordan. The potential impact of the reduction in forage availability is devastating and, eventually, livestock farmers depend on concentrates and grains as major sources of nutrients, which in turn decreases the profitability of raising sheep [2]. In this context, livestock producers have started to use alternative feeds and by-products in the diets of the ruminants [3,4], as a means to reduce the feed cost. In addition, it could potentially help to improve production efficiency by allowing farmers to better match herd demand with local feed resource availability and thereby decrease dependence on conventional diet ingredients. This may provide considerable economic benefits, knowing that feeds generally represent more than 70 percent of the ruminant rearing cost in Jordan [2]. Therefore, the use of alternative feeds in the livestock diets is increased due to the high cost of the conventional feeds; as a result, the use of alternative feeds will solve the problem of feeding sheep in Jordan [2].

Black cumin (*Nigella sativa*) is a small elegant annual herb that is used as a source of industrial seed oil and medicine. Black cumin is usually cropped for seed production [5] and claimed to contain natural products and secondary metabolites that could benefit humans and animals [6]. Black cumin meal (BCM), a by-product after oil extraction, is rich in nutrient content such as crude protein (330 g/kg), all essential amino acids, fat (127 g/kg) and energy [7], making it an excellent source of alternative ruminant feed. However, variations in the nutrient content of the BCM is noted, mainly the content of fat (53, 92 or 127 g/kg dry matter (DM)). The differences in nutrients are caused by the different methods used to extract the oil, such as using chemicals, cold extraction, or using heat. Therefore, it is recommended to analyze the nutrient content of the BCM before it is incorporated in the diets. Abdel-Magid et al. [8] reported that the BCM-containing diet enhanced growth performance of calves compared with the conventional diet. In another study, Mahmoud and Bendary [7] demonstrated that inclusion of BCM at 125 g/kg in the diet of lambs and calves did not affect dry matter intake, digestibility and growth performance. However, Mahmoud and Bendary [7] reported a positive effect of using BCM on the cost of diets for growing lambs and calves compared with free containing BCM diet. These previous studies showed that the inclusion of BCM is promising when used as alternative feed to supply crude protein. Therefore, the hypothesis of this study was that feeding with BCM during the growing period would not have a negative effect on the growth performance while increasing the profit of using such a product. Consequently, the aim of this experiment was to assess the influence of feeding with BCM on feed intake, nutrient digestibility, N balance and growth performance of growing lambs.

## 2. Material and Methods

All of the methods used in this experiment were approved via the Jordan University of Science and Technology (JUST) Institutional Animal Care and Use Committee (Protocol #: 16/03/03/275). Black cumin meal was purchased from a local oil company (Green Fields Oil Factory, Amman, Jordan) and transported to JUST campus and ground before it was mixed in the diet. A group of 24 male lambs were chosen at random from a group of 60 lambs, which were born in the animal farm at JUST. Before the commencement of the study, lambs were weighed, health checked and treated against internal parasites. At the beginning of the collection period, no difference in the initial body weight (BW) was observed between the two groups and the average was 16.9 ± 0.42 kg. Lambs were housed individually in shaded concrete-surfaced pens (1.5 × 0.75 m). Each pen was equipped with plastic waterers (7 L) and plastic feeders (10 L). Lambs had ad libitum access to diets (110% of the previous day’s intake) and water throughout the experiment.

Lambs (70 ± 4.52 days of age; mean ± SD) were randomly assigned to 1 of 2 iso-nitrogenous (160 g/kg crude protein (CP) of dietary DM) treatment diets. Diets were formulated to satisfy the nutrient requirements for growing lambs [9]. Diets were 0 g/kg BCM (CON; n = 12) or 150 g/kg BCM (BCM150; n = 12) of dietary DM (Table 1). The BCM was formulated into the BCM150 diet by partial replacement of barley grain and soybean meal (SBM). The price of each ingredient (cost/1000 kg) used in the diets was 332, 630, 70, 325, 155, 160, 770 US$ for barley grain, soybean meal, BCM, wheat straw, salt, limestone and vitamin-mineral premix, respectively. During the study, the diets were mixed once every two weeks and a sample was taken to analyze the chemical composition. The experiment period was 80 days, of which the first ten days were used to adapt animals to the diets and pens, followed by a period of seventy days to collect data. Feed intake was measured daily. Animals were weighed at the commencement of the study and every two-week period during the study.

For chemical composition determination, feed and refusals samples were dried in the oven at 50 °C to reach a fixed weight and then ground and kept for further analysis. The ground samples were analyzed for DM, CP, and either extract (EE) using AOAC [11] procedures. With adjustments for use in the ANKOM^2000^ fiber analyzer apparatus (ANKOM Technology Cooperation, Fairport, NY), neutral detergent fiber (NDF) and acid detergent fiber (ADF) were analyzed following the procedures of Van Soest et al. [12].

On day 49 of the experiment, 6 randomized animals (age, 118 ± 3.66 days; BW = 29.48 ± 2.95 kg; mean ± SD) were selected from each group and were placed in metabolic cages (1.05 × 0.80 m) to assess the digestibility of the nutrients and N balance. Animals were given five days to adapt to the crates and then five days to collect the data (i.e., feed intake and refusals, fecal and urine output). The metabolic cages were designed to collect fecal and urine output separately. The floor of the cages was designed to allow animal feces to pass through it so that there is a tray underneath it to collect the feces, as well as allow urine to pass into a plastic container located under the cage. Urine containers contained 50 mL 1 N HCL to prevent the losses of N during the collection period. During the collection period, ten percent of the feces and five percent of the urine were sampled daily and were kept (−20 °C) for chemical analysis. At the end of this period, all collected samples were composited for each lamb. Fecal samples were dried at 50 °C to reach a constant weight and ground to pass through a 1-mm sieve. Then, samples were analyzed for DM, CP, NDF, ADF and EE. Urine samples were analyzed for N (Kjeldahl procedure). Digestibility was calculated using the following equation:

Nutrient intake = (nutrient intake – fecal output) * 100%

nutrient intake

### Statistical Methods

The treatment diet was used as the only fixed effect and data had been analyzed using procedures of PROC MIXED of SAS [13]. Least square means were separated if the fixed effects were significant at (*p* ≤ 0.05).

## 3. Results

No health disorders or issues were observed among the animals during the experiment, indicating that feeding BCM at 150 g/kg DM is safe to be fed for growing lambs. The inclusion of BCM at 150 g/kg DM did not affect the nutrient content except for the EE, which was greater in the BCM compared with the CON diet (Table 2). The feed cost was higher for the CON diet than the BCM150 diet (Table 1).

The nutrients intake is shown in Table 2. Compared with the CON diet, feeding lambs BCM150 diet increased DM (*p* = 0.04), CP (*p* = 0.05), NDF (*p* = 0.01), ADF (*p* = 0.04), EE (*p* < 0.001) and metabolizable energy (ME) intake.

The digestibility of DM (*p* = 0.02), CP (*p* = 0.04) and EE (*p* = 0.002) was improved in lambs fed the BCM150 diet compared with the CON diet (Table 3). However, NDF (*p* = 0.13) and ADF (*p* = 0.83) digestibility did not differ between the two diets. Nitrogen intake was greater (*p* = 0.04) for lambs fed on the BCM150 diet compared with lambs fed on the CON diet. Nitrogen lost in feces (*p* = 0.98) and urine (*p* = 0.10) was not different between the two diets. However, N retained, g/d (*p* = 0.01) and N retention percentage (*p* = 0.05) was greater in the BCM150 diet compared with the CON diet.

Initial BW was similar (*p* = 0.16) between the two diets (Table 4). However, feeding BCM150 increased final BW (*p* = 0.002) and ADG (*p* = 0.001), improved feed efficiency (*p* = 0.01) and decreased the cost of gain (*p* < 0.001) compared with the CON diet.

## 4. Discussion

During the growing periods of lambs, the need for a balanced diet is the highest when compared with the other periods. Therefore, this critical period requires a well-balanced diet, which in general is considered very costly. To solve this problem, livestock producers have started to depend on alternative feeds as part of the diet [14], due to their price and good availability [15]. Consistent with the current study, no health problems were observed in other studies conducted in our laboratory using different alternative feeds (sesame meal, olive cake, bread by-products, dried distillers’ grains with solubles, carob pods, *Atriplex halimus* L.) [14,15,16,17]. Overall the nutrient content in the BCM (such as the CP and EE; Table 1) indicates how this ingredient to be used as an alternative feed. In the present study, the use of BCM at 150 g/kg of dietary DM did not affect most of the nutrients in the diets, with the exception of EE and metabolizable energy (ME) content, which was greater in the BCM150 diet vs CON diet. This is due to the fact that the content of EE is higher in the BCM compared with other ingredients. In harmony with the results obtained herein, dietary inclusion of BCM at 50 or 100 g/kg in sheep diets [18] or at 125 g/kg in growing lambs or at 70 and 110 g/kg in calves’ diets [19] increased EE content. The other nutrients (CP = 328 g/kg, NDF = 228 g/kg, ADF = 114 g/kg) contained in the BCM show how valuable this by-product is in feeding livestock, and that it can replace barley grain and soybean meal, the most expensive ingredients. These results are consistent with previous studies [7,20]. Mahmoud and Bendary [7] reported that CP and EE contents in the BCM were 331 and 127 g/kg DM, respectively. In addition, Thilakarathna et al. [20] showed that the CP and EE contents in the BCM were 189 and 159 g/kg DM, respectively. Based on the nutrient content of the diets reported herein, the use of BCM could potentially improve the capacity of sheep farming system in Jordan and the Mediterranean areas. However, due to the variations in nutrient content in BCM, it is recommended to analyze it chemically before the inclusion in the diets of sheep or small ruminants and it should be properly inserted.

The diminution in the cost of the diet (20%) in this experiment was due to reducing the level of barley (12%) and soybean meal in the BCM diets (45%) compared to the CON diet (Table 2). This was due to the low cost of BCM in comparison to the current price of barley grain and soybean meal, showing the economic advantage of feeding Awassi lambs diets containing BCM. Similarly, a beneficial impact was observed when BCM was included at 53 and 106 g/kg in diets of growing calves [8], when included at 125 g/kg in lambs and calves diet [7], when included at 350 g/kg in camel diet [21] or when included at 50 or 100 g/kg in rabbit diets [22]. In agreement, other studies reported the economic impact of using agro-industrial by-products in feeding livestock [3,14,23]. In addition to these beneficial effects, the use of BCM and other oil cakes as alternative feeds in livestock diet would diminish environment pollution compared to if they were wasted [4].

In the current study, the improved nutrient intake in lambs fed the BCM150 diet could be related to improved DM, CP and EE digestibility, N retention (Table 2) and/or a lower dustiness as a function of greater EE in the BCM150 diet vs the CON diet. Another explanation for these findings is due to the improvement in the gastrointestinal tract health [24], antimicrobial effects on the pathogenic microorganisms in the digestive system [25], to enhanced palatability [26] or improvement in immune system [19] or to reduced dustiness that was observed in the BCM150 diet compared with the CON diet. Enhanced nutrient intake in the current study is consistent with the observations of Abdel-Magid et al. [8] when BCM was fed to crossbred calves at 53 or 106 g/kg of dietary DM. In addition, Cherif et al. [5] reported a greater DM and other nutrient intakes for Barbarine lambs fed *Nigella sativa* seeds at 120 g/kg DM. Similarly, Habeeb and Tarabany [27] reported that DM intake increased in Zaraibi goats supplement with *Nigella sativa*. The improvement in nutrient DM and CP digestibility that was observed in the current study could be due to the fact that the environment of the rumen has been enhanced by the antimicrobial effects of BCM on the pathogenic microorganisms in the digestive system [25]. Consistent with our results, Abdel-Magid et al. [8] found that BCM had improved CP digestibility when included at 53 or 106 g/kg of dietary DM in the diets of calves. In this study, both groups had a positive N retention, which reflected positively on growth. However, the BCM group had better N retention than the CON group. This result can be explained by the increased CP digestibility that was observed in the BCM group compared with the CON group. Consistent with results obtained herein, Retnani et al. [26] observed more N retention in lambs fed BCM at either 100 or 200 g/kg. These findings indicate that the quality of CP obtained from BCM is high compared with other protein sources obtained from the ingredients.

In the current study, the two groups had comparable initial BW; however, final BW and ADG was greater in the BCM150 compared with the CON diet, indicating that lambs fed on the BCM150 were more efficient in converting nutrients to growth. It could be speculated that this result is due to the higher nutrient intake, mainly energy, which is considered to be one of the major factors affecting growth performance. In fact, the greater EE intake and digestibility in the BCM150 group provided more energy because the EE provides 2.25-fold more energy than either CP or carbohydrates. In addition, the higher N intake and CP digestibility of the BCM150 lambs could also have led to the improvement of N retention observed herein. Similarly, growth performance (final BW, total gain and ADG) was enhanced in growing calves fed BCM at 53 and 106 g/kg DM compared with the BCM-free containing diet [8]. Habeeb and Tarabany [27] reported that final BW and ADG improved in Zaraibi goats supplemented with *Nigella sativa*. Taha [18] and Retnani et al. [26] reported that the feeding of *Nigella sativa* cake at 50 or 100 and 100 or 200 g/kg in the diet of growing lambs increases BW and the average daily gain, respectively. In another study, Cherif et al. [5] reported a greater growth for Barbarine lambs provided diets with *Nigella sativa* seeds at 120 g/kg DM in low or high concentrate diets. In contrast to our finding, Mansour et al. [19] showed that final BW and total gain did not respond to different levels of BCM when fed to growing calves. The improvement in aforementioned growth performance data agreed with feed intake, nutrient digestibility and N balance data, indicating that BCM is a nutrient rich ingredient and could be used as part of the diets of growing lambs.

## 5. Conclusions

According to results obtained in the current study, it could be stated that feeding BCM to growing lambs improved the overall nutrient intake and digestibility, N retention, growth performance (final body weight, average daily gain, feed efficiency and cost of gain) parameters compared with the control diet. Therefore, BCM can be used as alternative to SBM and barley as the protein and energy supplements, respectively, in growing lambs’ diets. Moreover, more research studies are needed to study the effect of feeding BCM and different levels on carcass characteristics and meat quality of sheep or other livestock. However, due to the variability in nutrient content of BCM, it is recommended to be analyzed chemically before it is incorporated in the diets.

## Figures and Tables

**Table 1:** Ingredients and chemical composition of diets-containing black cumin meal (BCM) fed to Awassi lambs.

Item	Diet ^1^		
	CON	BCM150	BCM
Ingredients (g/kg DM)			
Barley grain, whole	500	440	
Soybean meal, 440 g/kg CP (solvent)	200	110	
Black cumin meal	0	150	
Wheat straw	280	280	
Salt	10	10	
Limestone	9	9	
Vitamin-mineral premix ^2^	1	1	
Nutrients (g/kg DM)			
Dry matter	915	924	918
Crude protein	161	163	328
Neutral detergent fiber	293	308	228
Acid detergent fiber	194	198	114
Ether extract	19	34	122
Metabolizable energy, Mcal/kg ^3^	2.31	2.70	5.34 ^4^

^1^ Diets were: the control diet (CON) or 150 g/kg BCM (BCM150) of dietary dry matter (DM). ^2^ Composition per kg contained (vitamin A, 600,000 IU; vitamin D3, 200,000 IU; vitamin E, 75 mg, vitamin K3, 200 mg; vitamin B1, 100 mg; vitamin B5, 500 mg; lysine 0.5%; DL-methionine, 0.15%; manganese oxide, 4000 mg; ferrous sulphate, 15,000 mg; zinc oxide, 7000; magnesium oxide, 4000 mg; potassium iodide, 80 mg; sodium selenite, 150 mg; copper sulphate, 100 mg; cobalt phosphate, 50 mg, dicalcium phosphate, 10,000 mg. ^3^ Estimated based on tabular values of NRC [10]. ^4^ Adapted from Gokdogan et al. [10].

**Table 2:** Effects of feeding with black cumin meal (BCM) on nutrient intakes of Awassi lambs.

Item	Diet ^1^			
	CON (n = 12)	BCM150 (n = 12)	SEM	*p* Value
Feed cost/ton (US$) ^2^	400	300	-	-
Nutrient intake, g/d				
Dry matter, g/d	1034	1124	30.4	0.04
Crude protein, g/d	167	183	6.9	0.05
Neutral detergent fiber, g/d	302	347	12.7	0.01
Acid detergent fiber, g/d	201	223	8.3	0.04
Ether extract, g/d	19	39	1.0	<0.001
Metabolizable energy, Mcal/d	2.39	3.03	0.103	0.0001

^1^ Diets were: the control diet (CON) or 150 g/kg BCM (BCM150) of dietary dry matter. ^2^ Calculated based on the prices of diet ingredients of the year 2019.

**Table 3:** Effects of feeding black cumin meal (BCM) on digestibility and N balance of Awassi lambs.

Item	Diet ^1^			
	CON (n = 6)	BCM150 (n = 6)	SEM	*p* Value
Digestibility, %				
Dry matter	75.2	78.9	0.74	0.024
Crude protein	73.6	78.1	1.09	0.041
Neutral detergent fiber	58.6	56.8	0.77	0.130
Acid detergent fiber	53.5	52.8	2.04	0.820
Ether extract	74.9	85.6	1.06	0.002
N balance				
N intake, g/d	31.4	34.2	0.64	0.035
N in feces, g/d	6.9	6.9	0.60	0.984
N in urine, g/d	9.7	7.8	0.64	0.104
N retained, g/d	14.8	19.5	0.58	0.005
Retention, g/100 g	47.5	57.0	2.41	0.048

^1^ Diets were: the control diet (CON) or 150 g/kg BCM (BCM150) of dietary dry matter.

**Table 4:** Effects of feeding black cumin meal (BCM) on growth performance of Awassi lambs.

Item	Diet ^1^			
	CON (n = 12)	BCM150 (n = 12)	SEM	*p* Value
Initial weight, kg	16.5	17.3	0.42	0.16
Final weight, kg	31.7	36.3	0.82	0.002
Average daily gain, g/d	217	272	8.4	0.001
Feed efficiency (DMI:ADG) ^2^	4.75	4.17	0.143	0.01
Cost/kg of gain (US$)	1.91	1.32	0.050	<0.001

^1^ Diets were: the control diet (CON) or 150 g/kg BCM (BCM150) of dietary dry matter. ^2^ DMI: ADG (dry matter intake:average daily gain).
